# Life-Threatening Hypokalemia Revealing CACNA1S-Related Hypokalemic Periodic Paralysis

**DOI:** 10.7759/cureus.106491

**Published:** 2026-04-05

**Authors:** Niyas Khalid Ottu Para, Seema Rab

**Affiliations:** 1 Internal Medicine, Burjeel Hospital Abu Dhabi, Abu Dhabi, ARE; 2 Internal Medicine, Burjeel Holdings, Abu Dhabi, ARE

**Keywords:** cacna1s mutation, calcium channelopathy, familial hypokalemic periodic paralysis, hypokalemic periodic paralysis (hypopp), severe hypokalemia

## Abstract

Familial hypokalemic periodic paralysis (HypoPP) is a rare autosomal dominant skeletal muscle channelopathy most commonly caused by pathogenic variants in the CACNA1S gene. It is characterized by recurrent episodes of severe hypokalemia and transient flaccid paralysis resulting from intracellular potassium redistribution rather than true potassium depletion. Despite advances in molecular genetics, familial HypoPP remains underrecognized, particularly in regions where acquired causes such as thyrotoxic periodic paralysis predominate.

We report a young adult male presenting with recurrent episodes of severe hypokalemia complicated by cardiac conduction abnormalities and requiring intensive care management. A systematic diagnostic evaluation, including next-generation sequencing, was undertaken to exclude secondary and renal causes of hypokalemia. Genetic analysis identified a heterozygous pathogenic CACNA1S variant (c.1583G>A; p.Arg528His), confirming hypokalemic periodic paralysis type 1 (HypoPP1).

This case highlights the importance of a structured diagnostic approach to recurrent hypokalemia and emphasizes the role of molecular testing in distinguishing familial channelopathies from more common acquired etiologies. We further discuss genotype-phenotype correlations, variable penetrance, implications for family screening, and emerging genotype-guided therapeutic strategies in hypokalemic periodic paralysis. The report underscores the growing importance of molecular diagnostics in the evaluation and management of rare neuromuscular channelopathies within the framework of contemporary precision medicine.

## Introduction

Periodic paralysis syndromes represent a heterogeneous group of inherited disorders of skeletal muscle excitability characterized by recurrent episodes of flaccid weakness in the absence of permanent neuromuscular deficits between attacks. These disorders belong to the broader category of skeletal muscle channelopathies, in which mutations affecting ion channels responsible for maintaining membrane excitability lead to episodic disturbances in muscle function. The periodic paralyses are traditionally classified according to the serum potassium level observed during paralytic attacks and include hypokalemic periodic paralysis, hyperkalemic periodic paralysis, and Andersen-Tawil syndrome, each associated with distinct genetic and electrophysiologic mechanisms [1-3].

Hypokalemic periodic paralysis (HypoPP) is the most prevalent form of periodic paralysis and is now firmly recognized as a primary skeletal muscle ion channel disorder rather than a disorder of potassium balance per se. The condition is typically inherited in an autosomal dominant pattern and is characterized by transient episodes of muscle weakness associated with marked reductions in serum potassium resulting from intracellular potassium redistribution. Episodes are frequently precipitated by metabolic or physiologic triggers such as high-carbohydrate intake, rest following strenuous exercise, infection, emotional stress, or dehydration [1-3].

Advances in molecular genetics have substantially improved understanding of the pathophysiology of HypoPP. Pathogenic variants in the CACNA1S gene, encoding the skeletal muscle L-type calcium channel CaV1.1, account for approximately two-thirds of genetically confirmed cases, while variants in SCN4A, encoding the skeletal muscle voltage-gated sodium channel NaV1.4, account for most of the remaining cases. These mutations typically affect the voltage-sensing domains of the ion channels and generate an abnormal gating pore current that destabilizes resting membrane potential and predisposes skeletal muscle fibers to paradoxical depolarization under conditions of reduced extracellular potassium [2,3]. This mechanistic insight has transformed HypoPP from a purely clinical diagnosis into a genetically defined skeletal muscle channelopathy.

Despite increasing recognition of its molecular basis, hypokalemic periodic paralysis remains underdiagnosed in many regions of the world [1,3]. In areas where secondary causes of hypokalemia, particularly thyrotoxic periodic paralysis, are more prevalent, inherited forms of the disorder may be overlooked or misclassified. Early recognition of characteristic clinical features, systematic exclusion of acquired causes, and timely molecular testing are therefore essential for establishing an accurate diagnosis and guiding appropriate therapy.

Genetic diagnosis carries important clinical implications. Beyond confirming disease etiology, identification of the causative mutation enables genotype-phenotype correlation, informs therapeutic decisions, and facilitates genetic counseling and family screening. Furthermore, growing insights into the molecular mechanisms of skeletal muscle channelopathies are opening avenues for targeted therapies aimed at correcting the underlying electrophysiologic defects.

Here, we report a case of recurrent life-threatening hypokalemia associated with genetically confirmed CACNA1S-related hypokalemic periodic paralysis. This case aims to describe a genetically confirmed case of CACNA1S-related hypokalemic periodic paralysis and to highlight the importance of a structured diagnostic approach, genotype-phenotype correlation, and mechanism-focused management in this condition.

## Case presentation

A young adult male presented to the emergency department with acute-onset generalized weakness that progressed over several hours, resulting in difficulty standing and walking. The weakness was preceded by sensory prodromal symptoms consisting of limb heaviness and paresthesia. On arrival, the patient was alert but markedly weak, with reduced muscle power involving all four limbs. There were no cranial nerve deficits, sensory loss, or sphincter disturbances. Deep tendon reflexes were diminished but present. Blood pressure was within normal limits, and there was no evidence of respiratory compromise.

**Figure 1:**
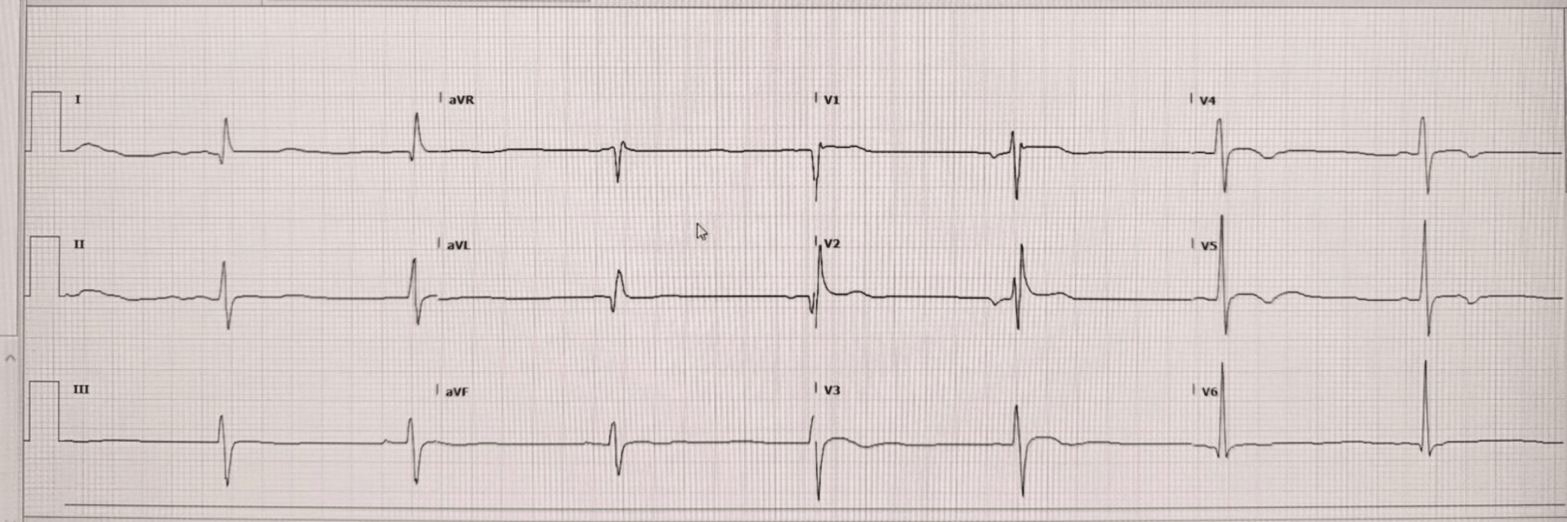
Electrocardiographic changes associated with severe hypokalemia Twelve-lead electrocardiogram demonstrating sinus bradycardia with repolarization abnormalities characterized by flattened T-waves and prominent U-waves, findings typical of profound hypokalemia. Such changes reflect delayed ventricular repolarization and increased arrhythmogenic risk during severe potassium depletion.

Initial laboratory evaluation revealed profound hypokalemia with a serum potassium level of 1.8 mmol/L (Table 1). Electrocardiography demonstrated marked sinus bradycardia with associated repolarization abnormalities, including flattened T-waves and prominent U-waves, findings consistent with severe potassium depletion (Figure 1). These findings raised concern for potentially life-threatening arrhythmias. Continuous cardiac monitoring was therefore initiated in the intensive care unit during controlled potassium replacement. Subsequent electrocardiograms demonstrated progressive normalization of cardiac conduction and repolarization abnormalities with potassium correction.

**Table 1:** Laboratory findings during the acute hypokalemic episode. Laboratory parameters obtained during the acute hypokalemic episode demonstrating severe hypokalemia with otherwise preserved renal function and no biochemical evidence of metabolic alkalosis. Abbreviations: eGFR, estimated glomerular filtration rate; CK, creatine kinase; CRP, C-reactive protein; TSH, thyroid-stimulating hormone; PRA, plasma renin activity.

Parameter	Value	Reference Range
Serum potassium	1.8 mmol/L	3.5 - 5.0 mmol/L
Sodium	143 mmol/L	135 - 145 mmol/L
Chloride	108 mmol/L	98 - 106 mmol/L
Bicarbonate	20 mmol/L	22 - 29 mmol/L
Creatinine	83 µmol/L	Normal
eGFR	>90 mL/min	Normal
Magnesium	0.915 mmol/L	0.7 - 1.0 mmol/L
Calcium	2.46 mmol/L	2.1 - 2.6 mmol/L
Phosphate	1.59 mmol/L	0.8 - 1.5 mmol/L
Creatine kinase (CK)	263 U/L	<200 U/L
C-reactive protein	0.3 mg/L	<5 mg/L
TSH	0.884 mIU/L	0.4 - 4.5 mIU/L
Plasma renin activity	4.65 ng/mL/hr	0.2 - 3.3 ng/mL/hr
Aldosterone	5.2 ng/dL	3 - 16 ng/dL
24-hour urinary potassium	62 mmol/day	25 - 125 mmol/24 hrs
24-hour urinary calcium	3.52 mmol/kg/day	2.50 - 8 mmol/kg/day

Venous blood gas analysis performed during intensive care monitoring demonstrated mild acidemia without evidence of metabolic alkalosis or lactic acidosis (Table 2). Electrolyte analysis revealed normal sodium levels with mildly elevated chloride and a reduced bicarbonate concentration. Lactate remained within normal limits. Overall, this biochemical pattern was not consistent with renal potassium-wasting disorders, which typically present with metabolic alkalosis.

**Table 2:** Venous blood gas and coagulation parameters during intensive care monitoring. Venous blood gas and coagulation profile obtained during the acute hypokalemic episode in the intensive care setting. Abbreviations: pH, potential of hydrogen; PT, prothrombin time; INR, international normalized ratio; aPTT, activated partial thromboplastin time.

Parameter	Value	Reference Range
Venous pH	7.33	7.35–7.45
Lactate	1.3 mmol/L	<2 mmol/L
Ionized calcium	1.10–1.14 mmol/L	1.10–1.30 mmol/L
PT	11.3 sec	Normal
INR	1.1	Normal
aPTT	31 sec	Normal

Additional biochemical evaluation demonstrated preserved renal function with a normal estimated glomerular filtration rate. Serum magnesium, calcium, and phosphate levels were within or near normal ranges. Creatine kinase was mildly elevated, likely reflecting transient skeletal muscle membrane instability during the paralytic episode rather than rhabdomyolysis. Inflammatory markers were not elevated.

Endocrine evaluation was undertaken to exclude secondary causes of hypokalemic paralysis. Thyroid function testing was within normal limits, effectively excluding thyrotoxic periodic paralysis. Assessment of the renin-aldosterone axis demonstrated elevated plasma renin activity with aldosterone levels remaining within the normal range, findings not consistent with primary hyperaldosteronism or other mineralocorticoid excess states.

Further investigation was performed to determine whether the hypokalemia resulted from renal potassium wasting or intracellular redistribution. Twenty-four-hour urinary potassium excretion measured 62 mmol/day, which was not inappropriate in the context of severe hypokalemia and did not support renal potassium-wasting disorders such as Bartter or Gitelman syndrome. Urinary calcium levels were within normal limits, and renal function remained preserved throughout hospitalization.

Intravenous potassium replacement was initiated in the emergency department and continued under monitored conditions in the intensive care unit. Gradual correction of serum potassium occurred over approximately 36 hours with serial electrolyte monitoring. As potassium levels normalized, the patient experienced progressive recovery of neuromuscular strength and resolution of electrocardiographic abnormalities. By the time of transfer from the intensive care unit to the general ward, muscle strength had returned to baseline, and cardiac rhythm had normalized.

The clinical course followed a characteristic sequence, beginning with acute presentation with severe hypokalemia and electrocardiographic changes, intensive care monitoring with controlled potassium replacement, progressive normalization of biochemical and electrophysiologic parameters over 36 hours, and complete recovery of neuromuscular function prior to discharge.

Further history obtained during hospitalization revealed that the patient had experienced two prior episodes of similar acute weakness, each associated with documented hypokalemia requiring medical evaluation. Between episodes, he remained asymptomatic with normal physical activity and exercise tolerance. Family history was notable for recurrent episodes of unexplained weakness in the patient’s mother, who reportedly experienced similar symptoms but died without a definitive diagnosis. A sibling was also reported to have experienced episodic weakness that had not undergone a formal medical evaluation. This pattern raised suspicion for a hereditary disorder with autosomal dominant inheritance and variable expressivity.

Given the episodic nature of paralysis, profound but reversible hypokalemia, absence of renal potassium wasting, normal thyroid function, and suggestive family history, a primary skeletal muscle channelopathy was suspected. Next-generation sequencing targeting genes associated with periodic paralysis and renal tubulopathies was therefore performed. Whole exome sequencing identified a heterozygous pathogenic variant in the CACNA1S gene (c.1583G>A; p.Arg528His), confirming the diagnosis of hypokalemic periodic paralysis type 1 (HypoPP1) as demonstrated in Table 3.

**Table 3:** Genetic confirmation of CACNA1S-related hypokalemic periodic paralysis Next-generation sequencing identified a heterozygous pathogenic missense variant in the CACNA1S gene (c.1583G>A; p.Arg528His), confirming the diagnosis of hypokalemic periodic paralysis type 1 (HypoPP1).

Category	Finding
Primary finding	CACNA1S p.R528H Missense Variant
Secondary findings	Negative
Interpretation	A heterozygous variant c.1583G>A (p.Arg528His) was identified in the CACNA1S gene, which is associated with hypokalemic periodic paralysis type 1 (HOKPP1).

Following stabilization, the patient was counselled regarding trigger avoidance, including prevention of dehydration, avoidance of excessive carbohydrate loads during recovery from illness, and early medical evaluation during episodes of weakness. The potential role of prophylactic pharmacologic therapy was discussed. Given the autosomal dominant inheritance pattern, family members were advised to undergo genetic counselling and screening.

## Discussion

This case illustrates the contemporary diagnostic framework for hypokalemic periodic paralysis, in which clinical chronology, biochemical physiology, electrophysiologic manifestations, and molecular genetics converge to establish a definitive diagnosis. The episodic nature of paralysis, complete inter-attack recovery, and absence of persistent biochemical derangements pointed toward a disorder of skeletal muscle membrane excitability rather than chronic potassium loss. The presence of profound hypokalemia accompanied by electrocardiographic repolarization abnormalities, followed by rapid recovery after potassium correction, further supported the diagnosis of a transient channelopathy-mediated process. Systematic exclusion of renal tubular disorders, mineralocorticoid excess states, and endocrine mimics was therefore essential in refining the diagnostic hypothesis prior to genetic confirmation.

Hypokalemic periodic paralysis is mechanistically distinct from renal and gastrointestinal causes of hypokalemia. In CACNA1S-related disease, pathogenic variants affecting the S4 voltage-sensing domains of the CaV1.1 channel create an aberrant gating pore current that permits inappropriate inward cation leak at resting membrane potentials. This results in paradoxical depolarization of skeletal muscle fibers, inactivation of voltage-gated sodium channels, and loss of muscle excitability. Physiologic stressors such as infection, carbohydrate load, insulin-mediated potassium shifts, rest following exertion, or dehydration exacerbate this vulnerability and precipitate paralytic attacks. Extracellular hypokalemia in this context reflects intracellular sequestration rather than true potassium depletion, explaining the profound yet rapidly reversible biochemical abnormalities observed during paralytic episodes [4-7].

Periodic paralysis syndromes comprise a heterogeneous group of inherited skeletal muscle channelopathies characterized by episodic weakness resulting from abnormalities in ion channel function. These disorders are broadly classified into hypokalemic periodic paralysis, hyperkalemic periodic paralysis, and Andersen-Tawil syndrome, each associated with distinct genetic mechanisms and clinical features. Hypokalemic periodic paralysis itself is genetically heterogeneous and is most commonly caused by pathogenic variants in CACNA1S (HypoPP type 1) or SCN4A (HypoPP type 2). CACNA1S-related disease accounts for approximately 60-70% of familial cases and typically presents with adolescent-onset episodic weakness triggered by metabolic or physiologic stressors. In contrast, SCN4A-associated HypoPP2 often demonstrates greater clinical variability and may overlap phenotypically with hyperkalemic periodic paralysis or paramyotonia congenita due to altered sodium channel gating. Andersen-Tawil syndrome, caused by variants in KCNJ2, represents a distinct entity characterized by the triad of periodic paralysis, ventricular arrhythmias, and dysmorphic skeletal features. Recognition of these genetic subtypes is clinically important because disease mechanisms, therapeutic responses, and long-term prognosis differ among the various channelopathies [4-8].

Although hypokalemic periodic paralysis is considered rare globally, its epidemiology varies considerably across geographic regions. In many Asian and Middle Eastern populations, a substantial proportion of hypokalemic paralysis cases are attributable to thyrotoxic periodic paralysis, an acquired disorder associated with hyperthyroidism. Familial hypokalemic periodic paralysis caused by ion channel mutations is reported less frequently in these regions and may therefore remain underrecognized. Consequently, patients presenting with recurrent hypokalemia are often initially evaluated for endocrine or renal causes, potentially delaying recognition of underlying skeletal muscle channelopathies [1-3]. The present case underscores the importance of maintaining clinical suspicion for inherited periodic paralysis when recurrent episodes occur in the absence of thyroid dysfunction or renal potassium-wasting disorders.

The CACNA1S c.1583G>A (p.Arg528His) variant identified in this patient represents one of the most extensively characterized pathogenic mutations associated with hypokalemic periodic paralysis type 1. Codon 528 lies within the S4 voltage-sensing segment of domain II of the CaV1.1 channel and constitutes a recognized mutational hotspot in HypoPP1. Arginine residues within the S4 region play a crucial role in voltage sensing due to their positive charge. Substitution of these residues disrupts normal gating behavior and produces an abnormal proton-selective gating pore current. Mutations affecting this residue, including p.Arg528His, p.Arg528Cys, and p.Arg528Gly, have repeatedly been identified in patients with familial hypokalemic periodic paralysis and are known to impair channel function through altered voltage sensing and abnormal membrane depolarization [5-7].

Despite the autosomal dominant inheritance pattern of HypoPP1, considerable variability exists in clinical expression even among individuals carrying identical mutations. Penetrance is incomplete and influenced by sex, hormonal factors, environmental triggers, and potential genetic modifiers. Some individuals experience frequent severe paralytic attacks beginning in adolescence, whereas others may remain mildly symptomatic or asymptomatic for prolonged periods [3,4]. Such variability may contribute to delayed diagnosis across generations, as illustrated by the family history observed in this case, where similar symptoms were reported in the patient’s mother but were never formally investigated.

Genotype-phenotype correlations have important therapeutic implications. Patients with CACNA1S-related HypoPP generally demonstrate favorable responses to carbonic anhydrase inhibitors such as acetazolamide and dichlorphenamide, which are believed to stabilize membrane excitability through mild metabolic acidosis and modulation of intracellular ion transport. In contrast, individuals with SCN4A-associated periodic paralysis may exhibit variable responses to acetazolamide, and some mutations have been associated with paradoxical worsening of weakness. In such cases, alternative strategies including potassium-sparing agents such as spironolactone, eplerenone, or triamterene may provide therapeutic benefit by minimizing potassium fluctuations and stabilizing membrane polarization. These genotype-dependent differences highlight the growing importance of molecular diagnosis in guiding individualized treatment strategies for periodic paralysis syndromes [8,9].

Dichlorphenamide has demonstrated efficacy in randomized controlled trials involving patients with periodic paralysis. Clinical studies have shown significant reductions in attack frequency and severity compared with placebo, supporting its use as an effective prophylactic therapy in patients with recurrent paralytic episodes. Importantly, treatment responses may vary according to the underlying genetic mutation, reinforcing the value of genotype-guided therapy in optimizing long-term disease control [8,9].

Beyond established therapies, emerging research is exploring interventions targeting the underlying pathophysiologic mechanisms of channelopathies. Experimental strategies aim to reduce gating pore currents or modulate sarcolemmal ion transport to restore membrane stability. Preclinical studies in animal models carrying CACNA1S mutations have demonstrated potential benefit from agents such as bumetanide, which may influence intracellular ion gradients and reduce abnormal membrane depolarization [7]. Although such therapies remain investigational, they illustrate the potential for mechanism-based treatment approaches in skeletal muscle channelopathies.

Another clinically relevant consideration is the association between CACNA1S mutations and susceptibility to malignant hyperthermia. The CaV1.1 channel interacts with the ryanodine receptor in skeletal muscle excitation-contraction coupling, and variants affecting this pathway may predispose individuals to malignant hyperthermia when exposed to triggering anesthetic agents [4,6]. Recognition of this association is important for perioperative management and underscores the value of genetic diagnosis in informing anesthetic risk.

The electrocardiographic abnormalities observed during the acute episode also highlight an important clinical aspect of hypokalemic periodic paralysis. Severe hypokalemia may produce significant cardiac conduction disturbances, including sinus bradycardia, repolarization abnormalities, and ventricular arrhythmias. Although these abnormalities typically resolve with correction of serum potassium levels, they underscore the potential for life-threatening complications during severe paralytic episodes and support the need for continuous cardiac monitoring during acute management [1].

Genetic counseling and family screening represent essential components of long-term management. Because HypoPP1 follows an autosomal dominant inheritance pattern, first-degree relatives have a 50% probability of carrying the pathogenic variant [1,3]. Identification of asymptomatic carriers allows anticipatory counseling regarding trigger avoidance, early recognition of paralytic episodes, and timely initiation of prophylactic therapy when appropriate.

From a broader clinical perspective, this case highlights the evolving role of genomic medicine in the evaluation of unexplained electrolyte disorders. Recurrent hypokalemia accompanied by episodic weakness often prompts extensive investigation for renal or endocrine etiologies. Integration of targeted genetic testing into the diagnostic pathway allows clinicians to identify underlying skeletal muscle channelopathies earlier and implement mechanism-directed management strategies. As genomic technologies become increasingly accessible, early molecular diagnosis will play a central role in the precision management of rare neuromuscular and electrolyte disorders.

Clinical learning points

Recurrent hypokalemia accompanied by episodic muscle weakness should prompt early consideration of skeletal muscle channelopathies, particularly when patients demonstrate complete recovery between attacks and when biochemical evaluation fails to identify renal potassium wasting or endocrine abnormalities.

Hypokalemic periodic paralysis is a disorder of skeletal muscle membrane excitability rather than true total body potassium depletion, characterized by intracellular potassium redistribution and transient muscle fiber depolarization during paralytic episodes. Genetic testing plays a crucial role in establishing a definitive diagnosis, as identification of pathogenic variants in genes such as CACNA1S or SCN4A not only confirms the underlying channelopathy but also informs long-term therapeutic strategies. Treatment responses may vary according to genotype, highlighting the importance of genotype-guided therapy in optimizing disease control.

Recognition of CACNA1S mutations has clinical implications beyond periodic paralysis itself, as variants affecting the CaV1.1 channel may confer susceptibility to malignant hyperthermia and increase the risk of cardiac conduction abnormalities during severe hypokalemic episodes, underscoring the importance of appropriate perioperative precautions and careful monitoring during acute attacks.

## Conclusions

This case highlights the importance of recognizing hypokalemic periodic paralysis as a disorder of skeletal muscle membrane excitability rather than a primary disturbance of potassium balance. The combination of recurrent episodic weakness, profound yet reversible hypokalemia, absence of renal potassium wasting, and a supportive family history should prompt early consideration of inherited skeletal muscle channelopathies.

Identification of the pathogenic CACNA1S c.1583G>A (p.Arg528His) variant confirmed the diagnosis of hypokalemic periodic paralysis type 1 and enabled a genotype-informed approach to management. Molecular confirmation not only clarifies the underlying disease mechanism but also plays a critical role in targeted therapy, risk stratification, and appropriate genetic counseling. As genomic testing becomes increasingly integrated into clinical practice, early genetic evaluation of unexplained recurrent hypokalemic paralysis can facilitate timely diagnosis, reduce the risk of life-threatening complications, and support individualized management of these rare but clinically significant skeletal muscle channelopathies.
